# Distribution and neurochemical characterization of neurons within the nucleus of the solitary tract responsive to serotonin agonist-induced hypophagia

**DOI:** 10.1016/j.bbr.2008.07.039

**Published:** 2009-01-03

**Authors:** Daniel D. Lam, Ligang Zhou, Andreas Vegge, Philip Y. Xiu, Britt T. Christensen, Mayowa A. Osundiji, Chen-yu Yueh, Mark L. Evans, Lora K. Heisler

**Affiliations:** a Department of Pharmacology, University of Cambridge, Tennis Court Road, Cambridge CB2 1PD, UK; b Department of Internal Medicine, Yale University School of Medicine, New Haven, CT 06520, USA; c Department of Veterinary Pathobiology, Faculty of Life Sciences, University of Copenhagen, Dyrlaegevej 88, 1870 Frederiksberg, Denmark; d Metabolic Research Laboratories, Institute of Metabolic Science, Addenbrooke’s Hospital, University of Cambridge, Cambridge CB2 2QQ, UK; e College of Medicine, Chang Gung University, Chang Gung Memorial Hospital, Chiayi, Taiwan, ROC

**Keywords:** Serotonin, mCPP, Nucleus of the solitary tract, Tyrosine hydroxylase, Catecholamine, Food intake, Rat

## Abstract

Pharmacological compounds enhancing serotonergic tone significantly decrease food intake and are among the most clinically efficacious treatments for obesity. However, the central mechanisms through which serotonergic compounds modulate feeding behavior have not been fully defined. The primary relay center receiving visceral gastrointestinal information in the central nervous system is the nucleus of the solitary tract (NTS) in the caudal brainstem. Here we investigated whether the classic anorectic serotonin receptor agonist *m*-chloro-phenylpiperazine (mCPP) enhances the activity of metabolically sensitive NTS neurons. Using *c-fos* immunoreactivity (FOS-IR) as a marker of neuronal activation in rats, we observed that mCPP significantly and dose-dependently activated a discrete population of caudal NTS neurons at the level of the area postrema (AP). In particular, this pattern of FOS-IR induction was consistent with the location of catecholamine-containing neurons. Dual-labeling performed with FOS-IR and the catecholamine biosynthetic enzyme tyrosine hydroxylase (TH) revealed that mCPP induced FOS-IR in 83.7% of TH-IR containing neurons in the NTS at the level of the AP. The degree of activation of TH neurons was strongly negatively correlated with food intake. Moreover, this activation was specific to catecholamine neurons, with negligible induction of cocaine- and amphetamine-regulated transcript (CART), cholecystokinin (CCK), glucagon-like peptide 1 (GLP-1), or neurotensin neurons. NTS catecholaminergic neurons relay visceral gastrointestinal signals to both the lateral hypothalamus (LHA) and paraventricular nucleus of the hypothalamus (PVH), where these signals are integrated into autonomic and hormonal responses regulating food intake. The data presented here identify a novel mechanism through which a serotonin receptor agonist acting in the caudal brainstem may regulate ingestive behavior.

Despite the rapidly increasing incidence of obesity, the mechanisms underlying the physiological control of feeding and body weight are only partially understood. The neurotransmitter serotonin (5-HT) is an important regulator of ingestive behavior. Depletion of brain serotonin levels produces hyperphagia and weight gain [3,27], whereas drugs which increase serotonin availability have an anorectic effect. One of the most extensively studied anorectic serotonin receptor (5-HTR) agonists is *m*-chloro-phenylpiperazine (mCPP), a compound that reduces food intake following peripheral and central administration [2,13,14,28]. mCPP decreases feeding rate and advances satiety in a similar manner to a food pre-load [15,32]. Thus, mCPP appears to reduce food intake by enhancing post-ingestive satiety.

Integral to the serotonergic regulation of food intake are serotonin neurons in the midbrain raphe nuclei that project to established hypothalamic feeding centers, including the arcuate (ARC) and paraventricular (PVH) hypothalamic nuclei. Specifically, serotonin engages melanocortin pathways in the ARC to inhibit feeding [9,10,16], while serotonin injected into the PVH has an anorectic effect [31].

In addition to the hypothalamus, the caudal brainstem also appears to be an important mediator of serotonergic effects on feeding. Chronically decerebrate rats, which lack direct neural connections between the hindbrain and forebrain, significantly reduce food intake in a manner comparable to intact rats when treated with peripheral doses of mCPP or d-fenfluramine, a drug which increases serotonin release and inhibits serotonin reuptake [8,12]. In addition, mCPP-induced hypophagia is blocked by fourth ventricle infusion of mesulergine, a 5-HT_2C/2A_R antagonist, providing indirect support for a brainstem locus of action [12]. In an effort to identify specific brain regions responsive to serotonergic hypophagia, surveys of associated c-fos immunoreactivity (FOS-IR) induction have been performed. Of note, anorectic doses of fenfluramine robustly increase neuronal activation in brainstem regions, including the nucleus of the solitary tract (NTS) [17,26].

The NTS is of particular interest because the majority of gustatory and gastrointestinal afferent pathways have their first synaptic relay in this region. Thus, NTS neurons are activated by visceral satietogenic signals such as gastric distension [22,34] and intestinal nutrients [36], as well as by meal ingestion [6,23]. Projections arising from the NTS relay gastrointestinal signals to hypothalamic regions involved in autonomic and hormonal control. In addition, second-order NTS neurons synapse on motor neurons in the dorsal motor nucleus of the vagus (DMV) which control ingestive and digestive function [21]. These connections represent a series of reflex pathways regulating eating rate and meal size. These reflex pathways are modulated by long-term energy signals both through direct effects of these signals in the caudal brainstem, and by reciprocal efferents from hypothalamic centers, where these long-term signals are integrated. For example, NTS neurons responsive to gastric vagal nerve stimulation are also activated by electrical stimulation of the PVH, a key feeding center [25].

In the present study, we investigated whether anorexia associated with serotonergic agonists like mCPP is correlated with the activation of a discrete population of NTS neurons. Specifically, we mapped FOS-IR induction produced by hypophagic doses of mCPP in the rostrocaudal extent of the NTS.

As a first step to investigate the central pathways involved in mCPP-induced hypophagia, we investigated the anorectic effect of mCPP at the onset of the dark cycle in *ad libitum* fed rats. All animal experiments were carried out in accordance with the “Principles of laboratory animal care” (NIH) and UK Home Office regulations. Experiments were carried out using adult male Sprague–Dawley rats weighing 280–300 g (Taconic), individually housed with water and rat chow pellets available *ad libitum* in a light-controlled (12 h on/12 h off) and temperature-controlled (21.5–22.5 °C) environment. Rats were implanted with a catheter in the femoral vein using methods previously described [4]. Five days later, rats received 0.9% saline or mCPP (Sigma, MO) dissolved in 0.9% saline (0.5, 2.5, or 5.0 mg/kg, i.v.; total volume injected = 0.25 ml; *n* = 4–5 rats per treatment). Rats were treated at the onset of the dark cycle and food intake was measured for the next 2 h. We observed that mCPP significantly and dose-dependently reduced chow intake compared to saline treatment (*F*(3, 17) = 7.59, *p* < 0.01; Fig. 1). Post hoc comparisons revealed that both 2.5 and 5.0 mg/kg of mCPP significantly decreased feeding compared to saline administration.

We next sought to investigate specific brainstem regions contributing to the anorectic effect of mCPP. Immunohistochemistry (IHC) for *c-fos* immunoreactivity (FOS-IR) was performed. Rats were deeply anesthetized with chloral hydrate (7%, 350 mg/kg, i.v.) and perfused transcardially with 0.9% saline followed by phosphate-buffed 10% formalin (pH 7.0, Sigma). Brains were removed, post-fixed with 10% formalin for 4 h and submerged overnight in 20% sucrose in phosphate-buffered saline (PBS). Brains were sectioned coronally on a freezing sliding microtome at 25 μm and collected in 6 equal series of adjacent tissue.

IHC was performed as previously described [4,5]. Briefly, tissue was pretreated in 0.3% H_2_O_2_ for 1 h to block endogenous peroxidase and then incubated with a rabbit anti-FOS antibody (1:10,000 concentration, Calbiochem) in 0.3% normal donkey serum and PBT (0.04% Triton X-100 in PBS) with 0.02% sodium azide (PBT-azide) overnight at room temperature. Next, sections were incubated for 1.5 h with biotinylated donkey anti-rabbit serum (1:500, Jackson Laboratories) in 0.3% normal donkey serum and PBT (0.04% Triton X-100 in PBS). Following this, sections were incubated with an avidin-peroxidase complex (ABC, Vector Elite kit; 1:250, Vector Laboratories) for 1 h in PBT. The immunoperoxidase was developed in 0.04% 3, 3′-diaminobenzidine tetrahydrochloride (DAB; Sigma) and 0.003% hydrogen peroxide in PBS. Each step was preceded by a 15 min PBS rinse. The DAB reaction was terminated within 6–12 min by rinsing with PBS. The sections were mounted onto polysine slides, air-dried, dehydrated in ascending concentrations of ethanol, cleared in xylene and cover-slipped with mounting medium (Micromount, Surgipath).

A qualitative survey of the distribution of FOS-IR in the NTS was performed (Fig. 2). Further quantitative analysis of FOS-IR induction was conducted at six selected levels of the NTS (Fig. 3), corresponding to −11.28 mm, −12.24 mm, −12.60 mm, −13.56 mm, −14.04 mm, and −15.00 mm from bregma based on the Paxinos and Watson rat brain atlas [20]. Cell counts were restricted to the NTS based on Nissl staining of adjacent sections. Cells were counted manually.

We observed that hypophagic doses of mCPP significantly and dose-dependently increased FOS-IR in the NTS only at levels −13.56 mm from bregma (*F*(3, 17) = 18.58, *p* < 0.0001; Fig. 3D) and −14.04 mm from bregma (*F*(3, 17) = 16.76, *p* < 0.0001; Fig. 3E). Post hoc analyses revealed that 0.5–5.0 mg/kg of mCPP significantly differed from saline treatment (Fig. S1, −14.04 mm from bregma displayed). Pearson product moment correlation analysis revealed a strong negative correlation between food intake and NTS FOS-IR at −14.04 mm from bregma (*r* = −0.85, *p* < 0.05). These data indicate that mCPP induces FOS-IR in a discrete subregion of the NTS, and that this activation is strongly negatively correlated with food intake.

Distributed in a pattern consistent with the FOS-IR induced by mCPP treatment is a cluster of catecholamine-containing neurons previously demonstrated to be responsive to nutritional status [23,24,34]. We therefore investigated whether mCPP significantly affects the activity of these neurons using dual-label IHC, with tyrosine hydroxilase immunoreactivity (TH-IR) used to identify catecholamine-containing neurons and FOS-IR as a marker of neuronal activation. Standard dual-label IHC procedures were used, as previously reported [35].

Briefly, tissue was rinsed with PBS for 15 min, then incubated with rabbit anti-c-fos (1:1000, Calbiochem) and mouse anti-tyrosine hydroxylase (1:1000, Chemicon) antibodies in 0.3% normal donkey serum and PBT-azide overnight at room temperature. Next, sections were incubated for 1 h with biotinylated donkey anti-mouse serum (1:500, Jackson Laboratories) in 0.3% normal donkey serum and PBT. Sections were then incubated for 1 h with appropriate fluorophores, Alexa fluor 594 conjugated to streptavidin (1:1000, Molecular Probes) and Alexa fluor 488 conjugated to donkey–anti-rabbit IgG (1:500, Molecular Probes). Finally, sections were mounted onto polysine slides, air-dried, and coverslipped with Vectashield mounting medium (Vector Laboratories). For dual-label IHC, the NTS at the level of the area postrema, between −13.56 mm and −14.04 mm from bregma, was analyzed. Only unequivocal perikarya were counted as positive for TH-IR and only unequivocal nuclei were counted as positive for FOS-IR.

mCPP significantly and substantially induced FOS-IR in TH-IR expressing neurons (Fig. 4B and D) compared to vehicle treatment (Fig. 4A and C) at −13.56 mm to −14.04 mm from bregma. Specifically, mCPP activated 83.7 ± 15.0% (mean ± S.E.M.) of TH-IR containing neurons, whereas saline treatment induced FOS-IR in less than 1% of TH-IR containing neurons. Pearson product moment correlation analysis revealed a strong negative correlation between food intake and proportion of NTS TH-IR neurons that contained FOS-IR at −14.04 mm from bregma (*r* = −0.89, *p* < 0.01). These data indicate that mCPP is highly effective in activating catecholamine-containing neurons in the NTS at the level of the area postrema, and that the extent of this activation is strongly negatively correlated with food intake.

To determine whether the activation of NTS catecholaminergic neurons is selective, colocalization of FOS-IR with populations of neurons in the NTS containing other neuropeptides with anorectic effects – cholecystokinin (CCK), cocaine- and amphetamine-regulated transcript (CART), neurotensin, and glucagon-like peptide 1 (GLP-1) – was assessed. The same dual-label approach was used with different primary antibodies (rabbit anti-CCK, Chemicon, 1:2000; rabbit anti-CART, Phoenix, 1:2000; rabbit anti-neurotensin, Immunostar, 1:2000; rabbit anti-GLP-1, Bachem, 1:2000; and/or goat-anti-c-fos, Santa Cruz, 1:1000).

CCK, CART, neurotensin, and GLP-1 containing neurons, were not activated by mCPP treatment, with less than 1% of each of these populations showing FOS-IR at any level of the NTS. The lack of coexpression of CART with c-fos after mCPP treatment is shown in Fig. S2, while corresponding results for neurotensin are shown in Fig. S3. Neither CCK nor GLP-1 are expressed at the levels of the NTS where c-fos induction is observed following mCPP, so no dual-labeling was observed with these neuropeptides (data not shown). Thus, the observed activation of NTS catecholeminergic neurons by mCPP is a selective effect.

The 5-HTRs most likely modulating mCPP’s effects on ingestive behavior in the NTS are the 5-HT_2C_Rs and/or 5-HT_1B_Rs since these receptors are critically involved in the serotonergic regulation of food intake and they are expressed in the caudal NTS [11,18,19]. However, mCPP also has binding affinity for the 5-HT_3_Rs, and activation of these receptors in the NTS may also play a role in mCPP hypophagia [7].

TH-containing neurons in the caudal NTS are evident in the mainly noradrenergic A2 cell group and, more rostrally, the mainly adrenergic C2 cell group [1,33]. These neurons appear to play an important role in the regulation of energy homeostasis. Gastric distension activates catecholaminergic neurons in the NTS [34] at a similar site to the maximal activation seen with mCPP. Specifically, 10–30% of NTS neurons positive for dopamine β-hydroxylase (DBH), which may be noradrenergic or adrenergic, were activated, depending on the degree of distension. In addition, satiating meals activated 25–35% of NTS TH neurons [23]. We performed neurochemical colocalization of TH-IR and mCPP-induced FOS-IR, and observed that the vast majority of TH-IR neurons in the NTS at the level of the area postrema, which includes A2, were activated by mCPP.

NTS TH neurons constitute a major ascending pathway through which vagal sensory information reaches the hypothalamus. In particular, neurons in the A2 region constitute one of the principal catecholaminergic inputs to the PVH and LHA [29,30]. Previous work in our laboratory has implicated hypothalamic centers in the anorectic actions of serotonin [9,10,16]. However, studies showing anorectic effects of mCPP and d-fenfluramine in decerebrate rats [8,12] suggest that serotonin can also modulate food intake by engaging brainstem receptors. Serotonin thus appears to affect food intake both at the level of reflex brainstem circuits and integrative hypothalamic systems. Further study is required to clarify these distinct actions and the relationship between them, and the role of NTS catecholaminergic neurons in either or both of these satiety networks.

In conclusion, we have shown that the classic serotonin receptor agonist mCPP selectively activates a specific subset of catecholaminergic neurons in the NTS at the level of the area postrema. These neurons play a key role in the integration of visceral satiety signals and may be involved in mediating brainstem reflex satiety circuits and/or in conveying visceral satiety signals to hypothalamic integration sites. The findings presented here provide additional insight into neural circuitry underlying the serotonergic modulation of ingestive behavior.

## Figures and Tables

**Fig. 1:**
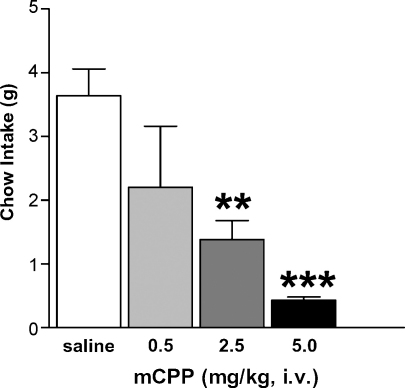
mCPP significantly and dose-dependently reduced 2 h food intake in rats. 0.9% saline (white bar) or mCPP at doses of 0.5 (light gray bar), 2.5 (dark gray bar), or 5.0 (black bar) mg/kg were administered i.v. at the onset of the dark cycle and food intake over the next 2 h was measured. Data are presented as mean ± S.E.M., **p < 0.01; ***p < 0.001 compared to saline treatment.

**Fig. 2:**
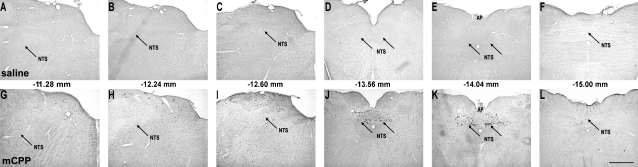
Distribution of mCPP-induced FOS-IR in the NTS. (A–F) 0.9% saline or (G-L) 5.0 mg/kg mCPP were administered i.v. at the onset of the dark cycle and 2 h later, rats were euthanized, brains extracted, prepared, and processed for FOS-IR. Displayed are representative photomicrographs of the nucleus of the solitary tract (NTS). Distances from bregma are (A and G) −11.28 mm, (B and H) −12.24 mm, (C and I) −12.60 mm, (D and J) −13.56 mm, (E and K) −14.04 mm and (F and L) −15.00 mm. mCPP selectively induced FOS-IR in a discrete region of the NTS (distance from bregma −13.56 mm to −14.40 mm) corresponding to the level of the area postrema (AP). Scale bar panel L, 1 mm, applies to (A)–(L).

**Fig. 3:**
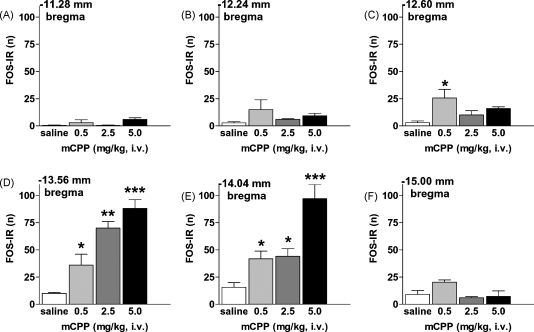
mCPP-hypophagia was associated with a significant induction of FOS-IR in the NTS. Counts of FOS-IR (n) induction in the NTS following treatment with 0.9% saline (white bar) or mCPP at doses of 0.5 (light gray bar), 2.5 (dark gray bar), or 5.0 (black bar) mg/kg, i.v. mCPP significantly increased FOS-IR in the NTS at −12.60 mm to −14.04 mm from bregma compared to saline treatment. Data are presented as mean ± S.E.M., *p < 0.05; **p < 0.01; ***p < 0.001 compared to saline treatment.

**Fig. 4:**
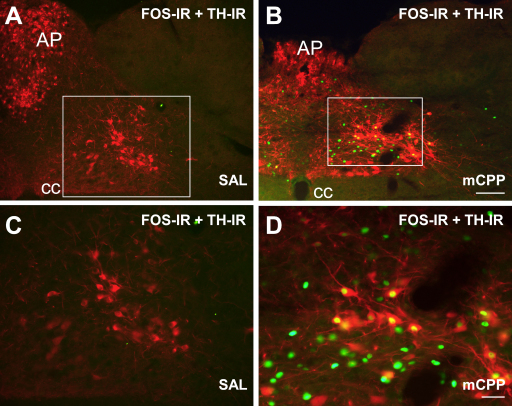
mCPP induced FOS-IR in NTS TH-IR neurons. Dual-label IHC for c-fos (green nuclei) and TH (red perikarya and fibers) was performed on tissue from rats treated with either 0.9% saline (SAL) or mCPP (2.5 mg/kg) (*n* = 4–5 per treatment). Merged photomicrographs of images of the individual fluorescent labels are shown. The images are from representative sections at the level of the area postrema (−13.56 mm to −14.04 mm from bregma). (A) Lack of c-fos expression in a rat treated with saline. (B) c-fos expression in NTS TH perikarya (yellow regions of colocalization) in a rat treated with 2.5 mg/kg mCPP. (C and D) Enlarged area represented in box in panels A and B, respectively. CC, central canal; AP, area postrema. Scale bar panel B, 100 μm, applies to (A) and (B); scale bar panel D, 20 μm, applies to (C) and (D).

## Appendix A Supplementary data

Fig. S1 mCPP dose-dependently induced FOS-IR in the NTS. (A and E) 0.9% saline, (B and F) 0.5 mg/kg mCPP, (C and G) 2.5 mg/kg mCPP, or (D and H) 5.0 mg/kg mCPP were administered i.v. at the onset of the dark cycle and 2 h later, rats were euthanized, brains extracted, prepared, and process for FOS-IR. At doses effective in reducing food intake, mCPP induced substantial and dose-dependent FOS-IR in a discrete region of the NTS corresponding to the level of the area postrema (distance from bregma −13.56 to −14.40). Displayed are representative photomicrographs of the NTS. E–H show enlarged areas represented in boxes in A–D. CC, central canal; AP, area postrema. Scale bar panel C, 1 mm applies to A–C; scale bar panel F, 100 μm applies to D–F.

Fig. S2 mCPP did not induce FOS-IR in NTS CART-IR neurons. Dual-label IHC for c-fos (green nuclei) and CART (red perikarya and fibers) was performed on tissue from rats treated with either 0.9% saline (SAL) or mCPP (2.5 mg/kg) (*n* = 4–5 per treatment). Merged photomicrographs of images of the individual fluorescent labels are shown. The images are from representative sections at the level of the area postrema (−13.56 mm to −14.04 mm from bregma). (A) Lack of c-fos expression in a rat treated with saline. Minimal CART-IR perikarya, but abundant CART-IR fibers, are found at this level of the NTS. (B) c-fos expression in NTS neurons, but not CART-IR perikarya, in a rat treated with 2.5 mg/kg mCPP. (C and D) Enlarged area represented in box in panels A and B, respectively. CC, central canal; AP, area postrema. Scale bar panel B, 100 μm, applies to (A) and (B); scale bar panel D, 20 μm, applies to (C) and (D).

Fig. S3 mCPP did not induce FOS-IR in NTS neurotensin-IR neurons. Dual-label IHC for c-fos (green nuclei) and neurotensin (NT, red perikarya and fibers) was performed on tissue from rats treated with either 0.9% saline (SAL) or mCPP (2.5 mg/kg) (*n* = 4–5 per treatment). Merged photomicrographs of images of the individual fluorescent labels are shown. The images are from representative sections at the level of the area postrema (−13.56 mm to −14.04 mm from bregma). (A) Lack of c-fos expression in a rat treated with saline. (B) c-fos expression in NTS neurons, but not neurotensin-IR perikarya, in a rat treated with 2.5 mg/kg mCPP. (C and D) Enlarged area represented in box in panels A and B, respectively. CC, central canal; AP, area postrema. Scale bar panel B, 100 μm, applies to (A) and (B); scale bar panel D, 20 μm, applies to (C) and (D).
