# Supplementary figures and images for: Distribution and neurochemical characterization of neurons within the nucleus of the solitary tract responsive to serotonin agonist-induced hypophagia
Source: Behav Brain Res. 2009 Jan 3;196(1):139–43. doi: 10.1016/j.bbr.2008.07.039 (PMC2614086; doi:10.1016/j.bbr.2008.07.039)

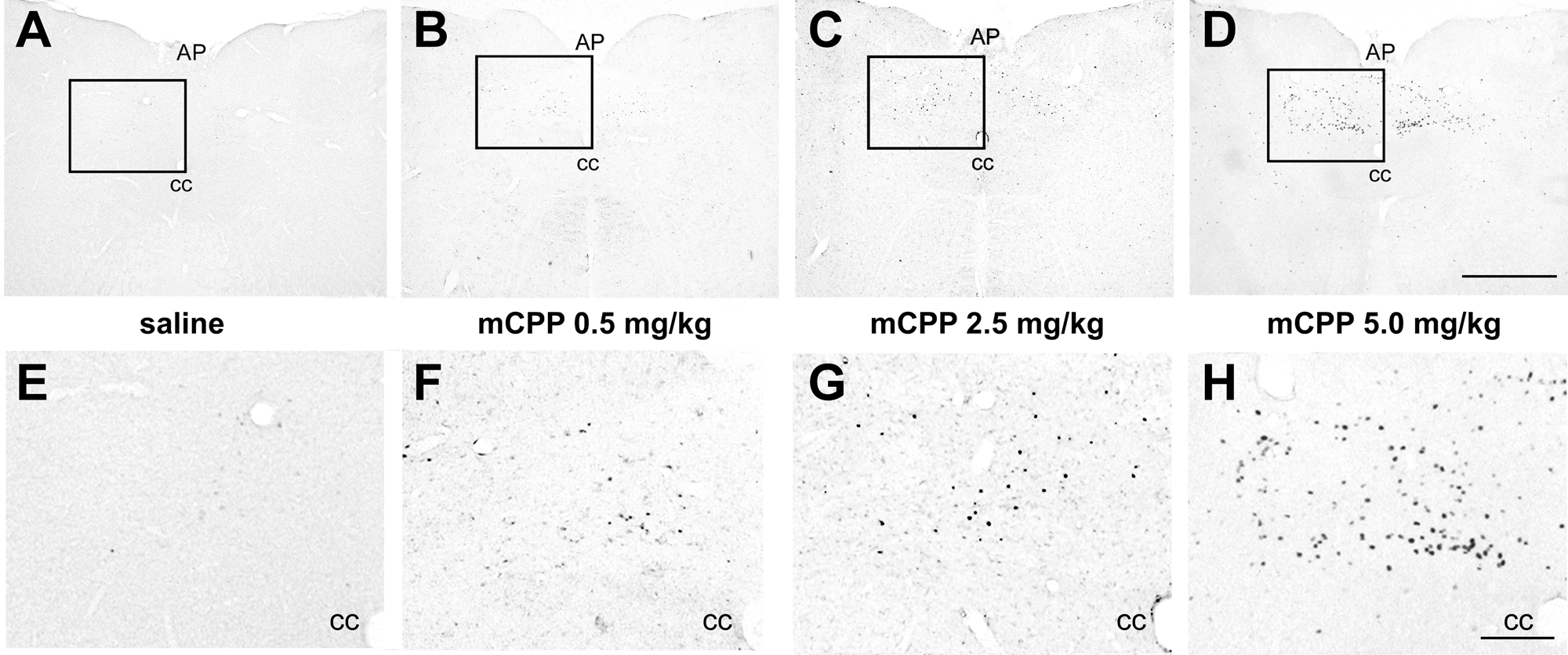

Supplement: Fig. S1 — mCPP dose-dependently induced FOS-IR in the NTS. (A and E) 0.9% saline, (B and F) 0.5 mg/kg mCPP, (C and G) 2.5 mg/kg mCPP, or (D and H) 5.0 mg/kg mCPP were administered i.v. at the onset of the dark cycle and 2 h later, rats were euthanized, brains extracted, prepared, and process for FOS-IR. At doses effective in reducing food intake, mCPP induced substantial and dose-dependent FOS-IR in a discrete region of the NTS corresponding to the level of the area postrema (distance from bregma −13.56 to −14.40). Displayed are representative photomicrographs of the NTS. E–H show enlarged areas represented in boxes in A–D. CC, central canal; AP, area postrema. Scale bar panel C, 1 mm applies to A–C; scale bar panel F, 100 μm applies to D–F. [file mmc1.jpg]

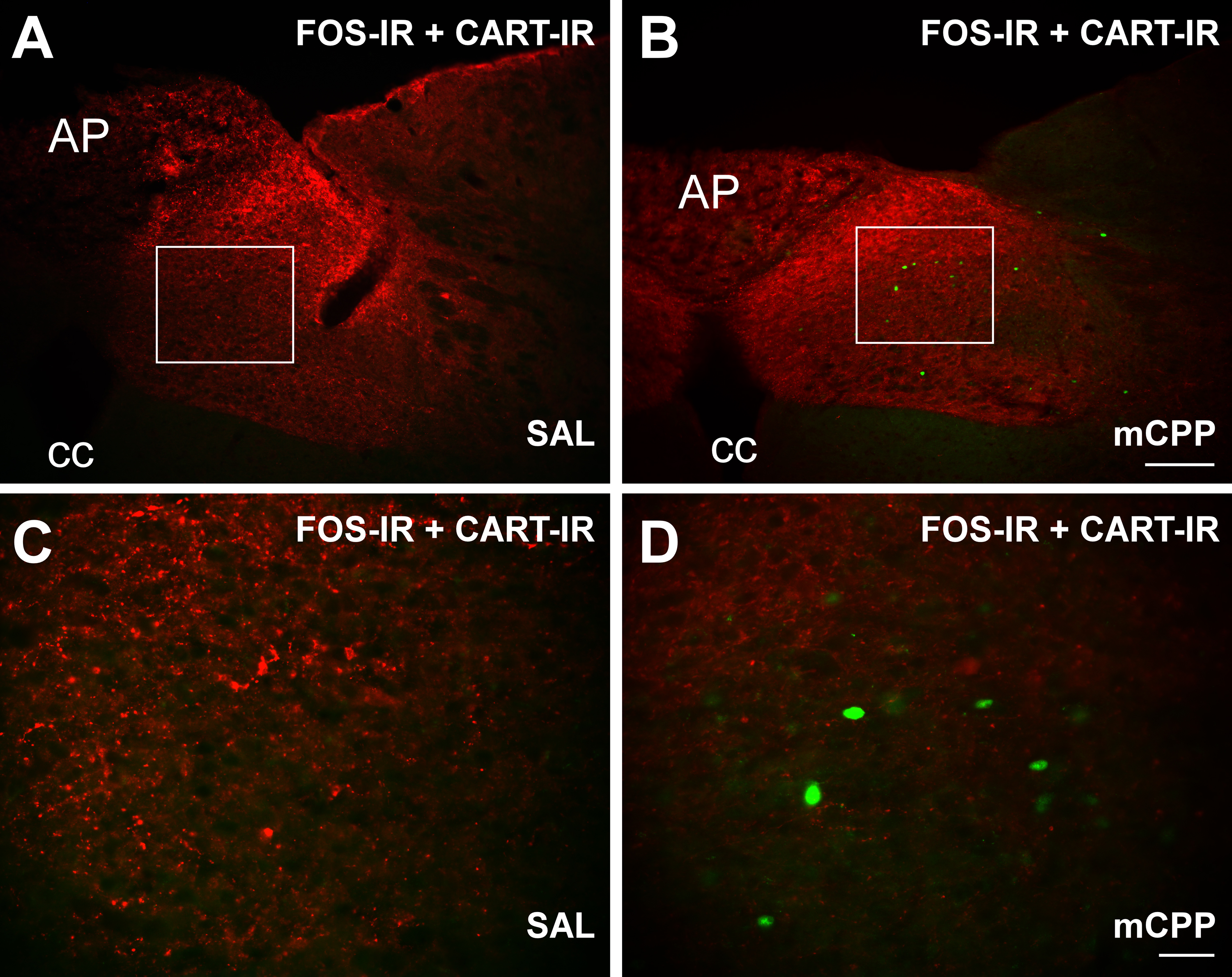

Supplement: Fig. S2 — mCPP did not induce FOS-IR in NTS CART-IR neurons. Dual-label IHC for c-fos (green nuclei) and CART (red perikarya and fibers) was performed on tissue from rats treated with either 0.9% saline (SAL) or mCPP (2.5 mg/kg) (n = 4–5 per treatment). Merged photomicrographs of images of the individual fluorescent labels are shown. The images are from representative sections at the level of the area postrema (−13.56 mm to −14.04 mm from bregma). (A) Lack of c-fos expression in a rat treated with saline. Minimal CART-IR perikarya, but abundant CART-IR fibers, are found at this level of the NTS. (B) c-fos expression in NTS neurons, but not CART-IR perikarya, in a rat treated with 2.5 mg/kg mCPP. (C and D) Enlarged area represented in box in panels A and B, respectively. CC, central canal; AP, area postrema. Scale bar panel B, 100 μm, applies to (A) and (B); scale bar panel D, 20 μm, applies to (C) and (D). [file mmc2.jpg]

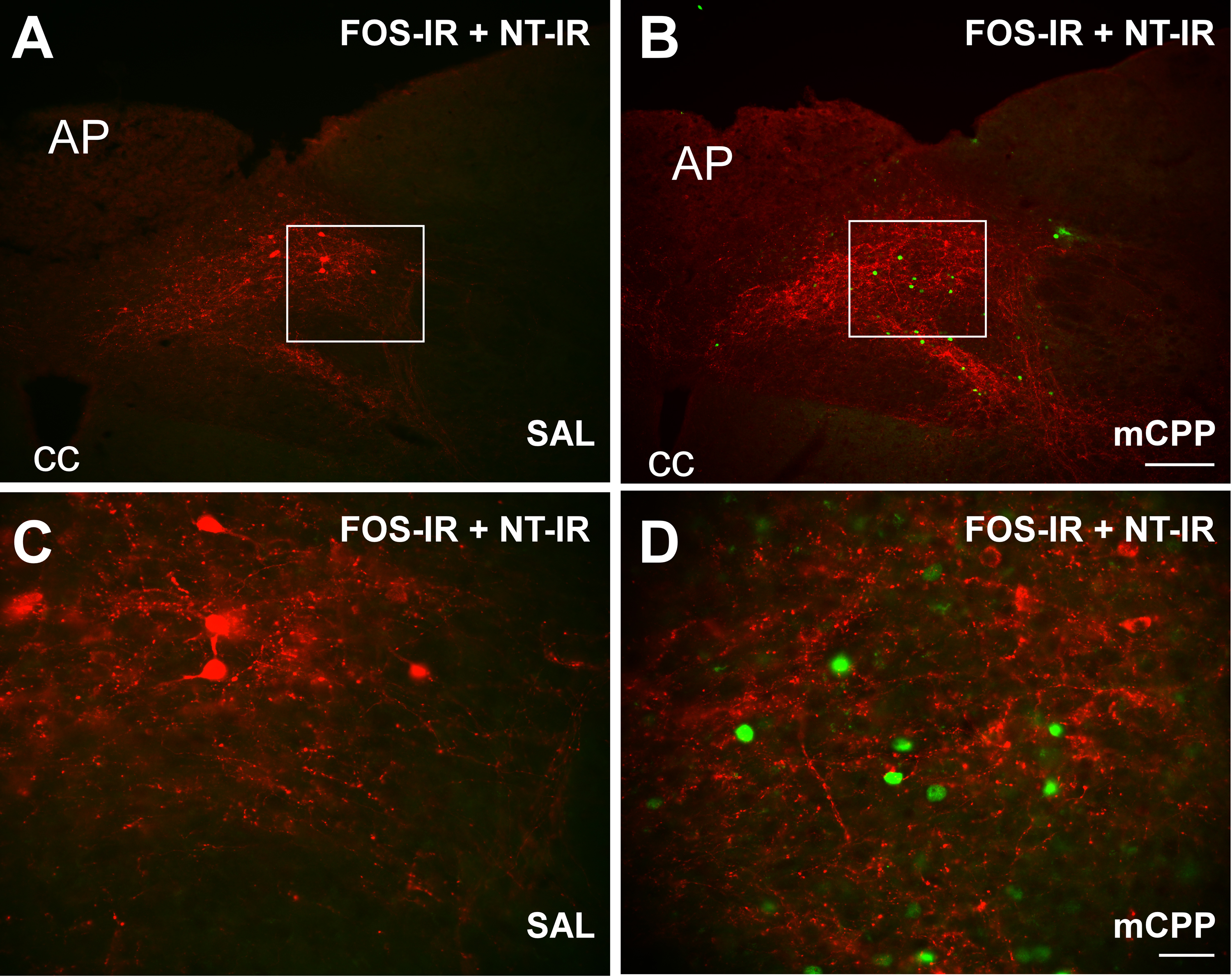

Supplement: Fig. S3 — mCPP did not induce FOS-IR in NTS neurotensin-IR neurons. Dual-label IHC for c-fos (green nuclei) and neurotensin (NT, red perikarya and fibers) was performed on tissue from rats treated with either 0.9% saline (SAL) or mCPP (2.5 mg/kg) (n = 4–5 per treatment). Merged photomicrographs of images of the individual fluorescent labels are shown. The images are from representative sections at the level of the area postrema (−13.56 mm to −14.04 mm from bregma). (A) Lack of c-fos expression in a rat treated with saline. (B) c-fos expression in NTS neurons, but not neurotensin-IR perikarya, in a rat treated with 2.5 mg/kg mCPP. (C and D) Enlarged area represented in box in panels A and B, respectively. CC, central canal; AP, area postrema. Scale bar panel B, 100 μm, applies to (A) and (B); scale bar panel D, 20 μm, applies to (C) and (D). [file mmc3.jpg]
